# Intrinsic and extrinsic goals as moderators of stress and depressive symptoms in Chinese undergraduate students: A multi-wave longitudinal study

**DOI:** 10.1186/s12888-016-0842-5

**Published:** 2016-05-11

**Authors:** Yu Ling, Yushu He, Yong Wei, Weihong Cen, Qi Zhou, Mingtian Zhong

**Affiliations:** College of Education, Hunan Agriculture University, Changsha, Hunan 410128 P. R. China; Business School, Central South University, Changsha, Hunan 410083 P. R. China; Center for Studies of Psychological Application, School of Psychology, South China Normal University, Guangzhou, 510631 P. R. China

**Keywords:** Undergraduate students, Intrinsic goals, Extrinsic goals, Stress, Depressive symptoms, Hierarchical linear model

## Abstract

**Background:**

Studies in western countries have examined the specific vulnerability hypothesis of Dykman’s theory of goal-orientation predispositions to depression through two-time point designs. The purpose of this prospective longitudinal study was to investigate the moderating effects of intrinsic and extrinsic goals on stress and depressive symptoms in Chinese undergraduate students.

**Methods:**

A total of 462 undergraduate students [46 % female; mean age, 19.06 (range, 17–22) years] completed self-reported measures assessing intrinsic and extrinsic goals, depressive symptoms, and the occurrence of social and academic hassles. Every 3 months over the subsequent 12 months, the undergraduate students completed measures assessing depressive symptoms and the occurrence of daily hassles.

**Results:**

Results of hierarchical linear modeling analyses indicated that undergraduate students with low levels of intrinsic goals reported greater depressive symptoms following the occurrence of social and academic hassles than did those with high levels of such goals. However, undergraduate students with high levels of extrinsic goals did not report greater depressive symptoms following the occurrence of social and academic hassles than did those possessing low levels.

**Conclusions:**

These findings suggest that intrinsic goals can protect undergraduate students experiencing high levels of social and academic hassles from depressive symptoms. The study findings provide new insight into the course of depressive symptoms among undergraduate students, and offer psychologist and psychiatrists ways to protect individuals from depressive symptoms by building up intrinsic goals.

## Background

Depression is the most serious mental health problem affecting undergraduate students, among whom rates of major depressive disorder range from 5.6 to 20 % [1–3]. Several recent studies have examined the prevalence of depression in university students worldwide [1, 4, 5]. Surveys conducted in the Chinese population have indicated that the prevalence of depressive symptoms among young Chinese undergraduate students ranges from 11.7 to 22.9 % [4, 6]. Depression in undergraduate students has been recognized as a major problem, as depressive symptoms are associated closely with suicidal behavior, substance abuse, college dropout, loss of academic productivity and antisocial behavior in this critical period of human development [7, 8]. Depression at college age also has a significant economic impact over the life course and serious consequences for later occupational trajectories [9].

Undergraduate students face a number of daily life stressors, and depressive symptoms have been shown to be closely related to various life events [10]. Problems that undergraduate students may face in this crucial stage, such as social competition, academic pressure, interpersonal may place them at high risk of developing negative effect (i.e., depressed mood) and, in serious cases, suicidal ideation and behavior [10, 11]. However, not all individuals who experience stressful life events develop depression, and a defense system can function to prevent this disease. The diathesis–stress framework posits that acute stressful life events and chronic stressful circumstances trigger an underlying predisposition, among the most promising explanations of how a depressive episode develops [12]. Such pre-existing vulnerability contributes to depressive symptoms and diagnoses only in the presence of life stress [13, 14]. However, the mechanism by which stress exerts this effect remains unclear.

Though the cognitive-diathesis-stress model is frequently used to explain people’s depressive symptoms, Dykman [15] listed three shortcomings of cognitive theories of depression and suggested these models lack “a vulnerability factor that is active or in motion prior to the occurrence of a negative event and that is capable of exerting a continuous influence on the depression-prone person’s functioning” (p.140). Shifting to the area of personal strivings and their relationships to motivated behavior, Dykman drew from the work of Dweck [16] and Eliott and Dweck [17], in which one’s implicit theories of learning and approach to a task determine subsequent engagement and achievement. He proposed a goal-orientation model of depression, positing that depression-prone and depression-resistant adults can be distinguished, at least in part, on the basis of whether they are primarily “validation seeking” (VS) or “growth seeking” (GS) in their personal strivings (or goal orientation). These scholars’ results supported the prediction that with increased stress, VS individuals would be more likely to experience depressive symptoms than GS individuals.

People’s goals vary. Self-determination theorists have distinguished intrinsic and extrinsic personal goals. Intrinsic goals, such as satisfying relationships, personal growth, and community contribution, are defined as pursuits that are generally congruent with the psychological needs for relatedness and autonomy [18–20]. Extrinsic goals, such as fame, attractiveness, and wealth, depend on the contingent reactions of others and are primarily concerned with obtaining some reward or social praise [20]. As a subtheory of self-determination theory, goal content theory distinguishes these two types of goal and examines their effects on behaviors and well-being [19, 21]. Extrinsic and intrinsic goals are thought to relate differently to basic need satisfaction and therefore to produce different psychological outcomes. Researchers have demonstrated that the prioritization of financial success over intrinsic goals (e.g., those related to community and family) is associated with decreased vitality, depression, and anxiety [22], whereas striving for intrinsic life goals is related to greater well-being [21, 23]. Relative intrinsic goal content has been found to positively predict physical self-worth, self-reported exercise behavior, psychological well-being, and psychological need satisfaction, and to negatively predict exercise anxiety [24].

There were two major limitations of previous research examining goal-orientation diathesis–stress models. First, the majority of previous research examining the specific vulnerability hypothesis of Dykman’s theory of goal-orientation predispositions to depression has relied on two-time point designs in which (a) goal orientation and depressive symptoms assessed initially and (b) depressive symptoms and negative events are assessed during follow up [15, 25]. Such a design necessitates the use of a nomothetic approach to the operationalization of high stress levels. Second, most previous prospective investigations of relationships between goal-orientation styles and depressive symptoms within a diathesis–stress framework have been conducted in Western cultural contexts. In a recent preliminary study conducted in Japan [25], researchers found that social learning goals reduced the effects of interpersonal stress, thereby protecting against depression. Social performance-avoidance goals, however, exacerbated the effects of interpersonal stress, hence facilitating thedevelopment of depression [25]. However, this study explored only social goal orientation, not general goal orientation.

The current study addresses these shortcomings by utilizing a multi-wave longitudinal design in which levels of stress and depressive symptoms were assessed at multiple time points (every 3 months) during a 12-month follow-up period and an idiographic approach to analysis. More specifically, we examined whether the slope of the relationship between negative events and depressive symptoms varied among undergraduate students as a function of levels of intrinsic and extrinsic goals. Consistent with the goal-orientation model and goal contents theory, lower intrinsic goals and higher extrinsic goals always related to higher level of depression [22], we hypothesized that (1) lower levels of intrinsic goals and (2) higher levels of extrinsic goals would be associated with greater within-subject increases in depressive symptoms following within-subject increases in stress levels.

## Method

### Participants

Participants were drawn from a five-wave longitudinal study of late-adolescent students from a university in Guangzhou, Guangdong, mainland China. The university was in urban setting, and all participants were living on campus. Only individuals who participated in at least two of five measurement waves [*n* = 472, 46 % female; mean age = 19.06 years, standard deviation (SD) = .89 years] were included in analyses. The ethnic distribution of these individuals (93.4 % Han, 6.6 % ethnic minority) is consistent with that in mainland China.

### Procedure

Prior to the initial assessment, consent forms that detailed the aims of the study were sent to potential participants. Undergraduate students were allowed to participate only if personal consent was received. A total of 553 students were asked to participate in the study, and 502 students returned their consent forms signed by themselves. Among the 502 students, only individuals who participated in at least two of five measurement waves were included in analyses. Finally, 472 students had effective data, and the effective response rate was 94 %.

The initial assessment was taken in September, 2013 and all of the assessments were taken during the free time after school. During the initial assessment, participants completed a demographic form and the following questionnaires: (1) the Center for Epidemiologic Studies Depression Scale (CES-D) [26], (2) the revised Aspiration Index (AI-R) [21], and (3) the General, Academic, and Social Hassles Scale for Students (GASHSS; Blankstein K, Flett G.Development of the general hassles scale for students.1993. Unpublished) [27]. Follow-up assessments were performed every 3 months for 12 months, during which participants completed the CES-D and GASHSS. It would take participants about 20 min to finish the initial assessment and about 10 min to finish each the follow-up assessment. Adolescents had just started their first year in college at the time of the first measurement wave, and were in their second year (if they had progressed with studies at a normative pace) at the time of the final measurement wave. The ethics committee of the South China Normal University approved the study, and all participants provided written informed consent before the survey began.

### Measurement

#### Depressive symptoms

The CES-D [26], a 20-item self-reported instrument, was used to assess students’ depressive symptoms. As an important screening tool for depressive symptoms, Center for Epidemiological Studies Depression Scale(CES-D) was widely used for assessing depressive symptoms exist among the normal and clinic sample from different age, gender and nationality [28–31]. Participants were asked to rate the occurrence of each symptom in the past week on a scale ranging from 1 (rarely) to 4 (most of the time). Total scores range from 20 to 80, with higher scores indicating higher levels of depressive symptoms. Scores of 36 or higher are considered indicative of depression [32]. The CES-D has been shown in numerous studies to have strong test-retest reliability and to be strongly correlated with other measures of depressive symptoms [33, 34]. The Chinese version of the CES-D has exhibited high degrees of reliability and validity [35]. In the current study, Cronbach’s alpha values for the CES-D ranged from 0.89 to 0.93 across administrations.

#### Intrinsic and extrinsic goals

Intrinsic and extrinsic goals were measured using the 35-item self-reported AI-R [21, 36]. Responses are structured by a scale ranging from 1 (not at all) to 7 (very), with higher scores reflecting greater orientation toward intrinsic and extrinsic goals. The intrinsic goal score is the sum of scores for the self-acceptance, affiliation, community feeling, and physical fitness subscales, and the extrinsic goal score is the sum of scores for the financial success, social recognition, and appealing appearance subscales. The AI-R has been shown to be reliable and to possess strong internal consistency [21, 37]. The Chinese version of the AI-R has also exhibited high degrees of reliability and validity [38]. In the present study, the Cronbach’s alpha value for the AI-R was 0.92.

#### Stress

Stress were evaluated using the abbreviated 30-item version of the GASHSS [27]. This instrument assesses general, academic, and social hassles. For each item, participants are asked to rate the frequency and duration of the hassle in the last 7 days (0 = no or not at all persistent hassle, 6 = extremely persistent or high-frequency and/or long duration hassle). Hassle scores range from 0 to 180, with higher scores reflecting a greater frequency of hassles. The Chinese version of the GASHSS has demonstrated strong internal consistency [39]. In the current study, Cronbach’s alpha values for the GASHSS ranged from 0.93 to 0.96 across administrations.

### Statistical analyses

Analyses were carried out using the SAS (version 9.0; SAS Institute, Cary, NC) MIXED procedure and maximum likelihood estimation. The dependent variable was within-subject fluctuations in depressive symptoms during the follow-up period, as predicted by goal-orientation style (intrinsic *vs.* extrinsic) and fluctuations in hassles. As goal-orientation style scores were between-subject predictors, they were standardized prior to analyses. As hassle was a within-subject predictor, measures were centered at each participant’s mean prior to analyses. Separate analyses were conducted for intrinsic and extrinsic goals. Preliminary analyses indicated that no reported association was moderated by age or gender; analyses are thus presented for the sample as a whole.

When fitting hierarchical linear models, appropriate mean and covariance structures must be specified. Covariance structures are commonly used in studies in which multiple responses are obtained from the same individual over time and include compound symmetry, first-order auto-regressive, heterogeneous autoregressive, and banded Toeplitz structures. To select a covariance structure for our analyses, we fitted the models utilizing each structure and chose the best fit based on standard and corrected Akaike information criteria and the Schwarz Bayesian information criterion. In all cases, the best fit was an autoregressive heterogeneous (ARH) structure.

After choosing the appropriate covariance structure, we next examined the random-effects component of our model. According to Diggle, Heagerty, Liang, and Zeger’s (2002) recommendation to use a “saturated” model for mean structure while searching for an appropriate covariance structure, we chose a mean structure that included intrinsic goals, hassles, and intrinsic goals × hassles interaction. Four additional effects were also included in this initial mean structure. First, as levels of depressive symptom likely differ among participants experiencing average individual levels of hassles, a random intercept was included in the model. Second, given that depressive symptoms are a within-subject predictor whose effect is expected to vary among participants, a random effect for slope was included in the model. Third, to control for individual differences in baseline levels of depressive symptoms, participants’ initial depressive symptoms were included in the model. Finally, given that hassle is a within-subject predictor, a random slope for hassles was also inserted into the model.

## Results

### Descriptive statistics

Means and standard deviations of depressive symptom and hassle measures obtained at T1–T5 are presented in Table 1. Although CES-D scores were higher at the T3 than at other time points, assessment time point had no significant main effect on CES-D scores [*F*(4,2146) = .61, *p* = .656]. Assessment time point had a significant main effect on GASHSS scores [*F*(4,2141) = 30.13, *p* < .001], which were significantly higher at the initial assessment [Student-Newman-Keuls (SNK) tests, *p* < .001)], and significantly lower at the follow-up 4 assessment (SNK test, *p* < .05) than at other time points.

**Table 1:** Observed means and standard deviations of depressive symptoms and hassles

	CES-D		GASHSS	
	Mean	SD	Mean	SD
Time 1	29.499	7.631	92.122	26.987
Time 2	29.757	7.725	78.423	27.211
Time 3	30.261	8.272	79.248	28.490
Time 4	29.685	7.881	76.674	29.707
Time 5	29.608	8.625	72.286	30.961

Note. *CES-D* Central for Epidemiological Studies Depression Scale, *GSAHSS* General Social and Academic Hassles Scale for students

Pearson correlations between all initial measures are presented in Table 2. Higher levels of extrinsic goals were associated with higher levels of depressive symptoms and more frequent occurrence of hassles, whereas no significant correlation was observed between these factors and intrinsic goals.

**Table 2:** Intercorrelations amongst initial CES-D, GSAHS and AI

	Initial CES-D	Initial GSAHSS	AI-Intrinsic goals
Initial CES-D	1		
Initial GSAHSS	.23***	1	
AI-Intrinsic goals	-.03	.07	1
AI-Extrinsic goals	.10*	.21***	.49***

Note. *CES-D* Central for Epidemiological Studies Depression Scale, *GSAHSS* General Social and Academic Hassles Scale for students, *AI* Aspiration Index

^*^ *p* <0.05; ^***^ *p* <0.001

### Intrinsic goals predicting depressive symptoms

When the random-effects component of the “saturated” model was examined, non-significant random effects were removed from the model prior to examination of the fixed-effects component. The ARH parameter (*r* = 2.46, *p* < .05), random intercept (*r* = 8.80, *p* < .001), and random slope (*r* = 9.81, *p* < .001) were significant and thus retained in the model. Table 3 shows covariance parameter estimates for the final model. The fixed-effects component of the model showed no significant main effect of intrinsic goals [*b* = –.048, standard error (SE) = .246, *F*(1,458) = –.20, *p* = .845] and significant two-way, cross-level interaction between intrinsic goals and stress [*b* = –.022, SE = .010, *F*(1,1175) = –2.30, *p* < .05].

**Table 3:** Estimates of the fixed effects component of the simultaneous model: hassles* intrinsic goals → depressive symptoms

Predictor	Parameter estimate(b)	SE	t value	df
Initial CES-D	4.580	.246	18.62***	1,458
GSAHSS	.043	.010	4.31***	1,1175
AI-Intrinsic goals	-.048	.246	-.20	1,458
GSAHS*AI-intrinsic goals	-.022	.010	−2.30*	1,1175

Note. *CES-D* Central for Epidemiological Studies Depression Scale, *GSAHS* General Social and Academic Hassles Scale for students, *AI* Aspiration Index

^*^ *p* <0.05; ^***^ *p* <0.001

**Fig. 1:**
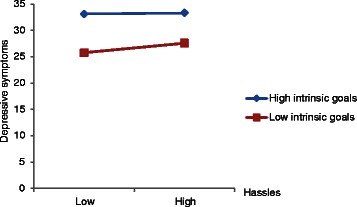
Intrinsic goals and stress as predictors of depressive symptoms

The fixed effects summarized in Table 3 were used to calculate predicted depressive symptoms for participants with high and low levels of intrinsic goals (±1.5 × mean between-subject standard deviation). Participants with low levels of intrinsic goals reported higher levels of depressive symptoms when experiencing high levels of hassles than when experiencing low levels of hassles [*b* = .076, SE = .018, *t*(1175) = 4.17, *p* < .001; Fig. 1]. However, levels of depressive symptoms did not vary as a function of high levels of intrinsic goals [*b* = .010, SE = .017, *t*(1175) = .60, *p* = .546). The slope of the relationship between hassles and depressive symptoms was significantly greater in participants with low than in those with high levels of intrinsic goals [*b* = –.066, SE = .029, *t*(1175) = –2.30, *p* < .05].

### Extrinsic goals predicting depressive symptoms

Similar analyses were conducted to examine whether students with extrinsic goals exhibited greater elevations in depressive symptoms following increases in hassles. The ARH parameter(*r* = 2.31, *p* < .05), random intercept (*r* = 8.78, *p* < .001), and random slope (*r* = 9.69, *p* < .001) were significant and thus retained in the model. In the first model, we estimated the effects of extrinsic goals on the relationship between hassles and depressive symptoms. Extrinsic goals exhibited no significant main effect on depressive symptoms [*b* = .231, SE = .247, *F*(1,460) = .93, *p* = .351] and no significant cross-level interaction between extrinsic goals and hassles was observed [*b* = –.006, SE = .010, *F*(1,1179) = –.57, *p* = .571].

## Discussion

In this study, we focused on the moderating roles that intrinsic and extrinsic goals can have on hassles and depressive symptom development among undergraduate students using a five-wave longitudinal design with 3-monthly assessments. First, in line with a previous study [40] in which stress was found to be highest at the initial assessment, our findings indicate that the level of hassles was highest at the time of the first assessment and declined gradually over time. These results likely reflect practice effects due to repeated administration of measures. Additionally, it likely reflects the major transition of beginning college. Stress and depression levels may decrease as students acclimate to college life.

Second, consistent with our hypotheses, lower levels of intrinsic goals were associated with greater increases in depressive symptoms following the occurrence of hassles. The finding that intrinsic goals might protect undergraduate students from depressive symptoms in the presence of hassles is consistent with Dykman’s conceptualization of the association between intrinsic goals and depressive symptoms within a diathesis–stress framework [15]. Kuroda and Sakurai obtained similar results indicating that social learning goals reduced the effects of interpersonal stress, thereby protecting against depression [25]. Sideridis argued that mastery-oriented individuals’ (those with intrinsic goals) engagement in a task arises from inherent needs for growth, learning, and improvement and is a consequence of secure attachment [41]. Undergraduate students pursuing these goals are thus more likely to show adaptive cognitive, affective, and behavioral patterns in the face of all kinds of hassle. Even in the case of failure, intrinsic goal–oriented undergraduate students appraise a given situation as an opportunity to learn and grow from their mistakes. In other words, they are motivated to interpret negative events as opportunities for self-growth. Such functioning buffers against the adverse effects of hassles, thereby aiding in the protection against the onsets of depressive symptoms. Fostering the development of intrinsic goals in prevention and intervention programs may, ultimately, help to prevent the onset and maintenance of depressive disorders.

Finally, contrary to our hypothesis, undergraduate students with higher levels of extrinsic goals did not report greater increases in depressive symptoms following the occurrence of hassles relative to those with low extrinsic goals. Researchers found that social performance-avoidance goals, a specific form of extrinsic goal, exacerbated the effects of interpersonal stress, thereby leading to the development of depression; however, they also found that social performance-approach goals (another form of extrinsic goal) did not have the same effect [25]. For undergraduate students pursuing extrinsic goals such as fame, attractiveness, and wealth, the occurrence of hassles implies that they have failed to attain their goals. They are thus threatened by such hassles. However, extrinsic goals may have different meanings for Chinese and Western undergraduate students. Previous researchers examining the life goals of undergraduate students in 15 countries found that intrinsic and extrinsic life goals opposed each other, but that extrinsic goals were grouped more with safety goals in poorer compared with wealthier countries, indicating that they have different meanings for individuals in these countries [42]. This finding has been confirmed by other researchers, who have found no clear relationship between wealth and happiness once basic human needs have been met [43]. Thus, because Chinese undergraduate students pursuing extrinsic goals focus on potential positive social outcomes (i.e., positive evaluations from others; valence hypothesis), they are more likely to show adaptive cognitive, affective, and behavioral patterns after the occurrence of hassles. For example, they are motivated not to focus on past negative evaluations, but to anticipate positive evaluations from others. Furthermore, they are more likely to utilize active and relatively adaptive coping strategies to deal with hassles in order to attain positive social outcomes in the future. Overall, extrinsic goals play both negative and positive roles in the face of hassles, which might explain why depressive symptoms among adolescents cannot be predicted on the basis of interactions between extrinsic goals and hassles.

Collectively, these findings advance our knowledge on the roles of intrinsic and extrinsic goals in the development of depressive symptoms following the occurrence of hassles. More specifically, the occurrence of negative events was associated with increases in depressive symptoms, but only among those with low levels of intrinsic goals. This result suggests that high levels of intrinsic goals might buffer against the deleterious effect of hassles on depression. Such findings highlight the importance of intrinsic goal orientation for the prevention and treatment of depression [44]. Intrinsic goal–oriented approaches to intervention focus on the cultivation of intrinsic goals such as self-acceptance, affiliation, community feeling, and physical fitness. Such interventions are likely to increase the quality and degree of goal orientation, thereby providing individuals with better resources to cope with the stressors they face and consequently avoid depression.

The present study implemented several empirical and methodological improvements over previous studies of intrinsic and extrinsic goals. First, it was the first study to examine the roles of intrinsic and extrinsic aspirations among Chinese undergraduate students using a diathesis–stress framework. Second, the study utilized a multi-wave design with repeated measures of stressors and psychological symptoms. In contrast to many cross-sectional studies of goal orientation, the use of a longitudinal design enabled the examination of temporal change in symptoms of depression and idiographic analysis of the diathesis–stress hypotheses. Third, this study extends knowledge on diathesis–stress models of depression in Chinese undergraduate students.

Several limitations of the study should be noted to provide direction for future research. First, the study utilized self-reported measures, which are prone to response biases and have diagnostic limitations. Future research should continue to explore how aspirations shape subsequent psychopathology by utilizing more sophisticated assessment techniques, such as peer and parent ratings, semi-structured diagnostic interviews, and direct behavioral observation, to examine these constructs. Second, the homogeneity of the sample of undergraduate students may limit the generalizability of our results to community or clinical populations. Additional research is warranted to determine whether the present results can be replicated in such populations. Third, as stress is one of the most robust predictors of psychopathology [45], other types of stressors are also likely to shape depressive symptoms. Further, the current study did not identify specific negative life events that may have exhibited particularly robust associations with depressive symptoms. Thus, future research is warranted to examine whether different types of stressors differentially predict psychopathology and whether specific life events may be differentially predictive of depression.

## Conclusions

The results of the current study provide support for the hypothesis that intrinsic goals serve as a protective factor in the development of depressive symptoms in a sample of Chinese undergraduate students. The study also provides further support for the applicability of the diathesis–stress model of depression to Chinese undergraduate students, suggesting that diathesis–stress processes predicting depressive symptoms may be universal, rather than culturally specific. In summary, intrinsic goals may protect Chinese individuals against the development of depression; thus, fostering the development of intrinsic goals in prevention and intervention programs may, ultimately, help to prevent the onset and maintenance of depressive disorders.

### Ethics

This study has been approved by the ethics committee of South China Normal University.

### Consent to participate

Each subject has signed the written informed consent before participation.

### Consent to publish

All subjects provided written consent for the publications of anonymized individual data.

### Availability of data and materials

In accordance with subjects’ informed consent, data and materials supporting our findings in the manuscript will not be shared.

## Abbreviations

AI: Aspiration Index
AI-R: the revised Aspiration Index
ARH: autoregressive heterogeneous
CES-D: Center for Epidemiologic Studies Depression Scale
GASHSS: the General, Academic, and Social Hassles Scale for Students
GS: growth seeking
SD: standard deviation
SE: standard error
SNK: Student-Newman-Keuls
VS: validation seeking
